# A modified adrenal gland-sparing surgery based on retroperitoneal laparoscopic radical nephrectomy

**DOI:** 10.1186/1477-7819-12-179

**Published:** 2014-06-05

**Authors:** Zhenyu Xu, Zhengyu Zhang, Jianping Gao, Zhifeng Wei, Xiaofeng Xu, Jie Dong, Hao Tang, Xiaoming Yi, Chaopeng Tang, Wenquan Zhou

**Affiliations:** 1 Department of Urology, Jinling Hospital, Medical School of Nanjing University, 305# East Zhongshan Road, Nanjing 210002, China

**Keywords:** renal neoplasms, retroperitoneal laparoscopy radical nephrectomy, adrenal gland-sparing, anatomy

## Abstract

**Background:**

The objective of this study was to modify the adrenal gland-sparing strategy based on retroperitoneal laparoscopic radical nephrectomy by reviewing the anatomic relationship between the kidney and the adrenal gland.

**Methods:**

From June 2010 to October 2012, a total of 68 patients (45 males and 23 females) with localized renal cell carcinoma were treated at our hospital. The study included 35 cases that were right side and 33 cases that were left, and average patient age was 54.06 years. The average tumor size was 4.7 cm. Tumors were classified via the TNM staging system. All patients underwent adrenal gland-sparing surgery based on retroperitoneal laparoscopic radical nephrectomy.

**Results:**

For each patient, surgery was successful without conversion to open surgery. The average operative time was 56.65 ± 26.60 min, and the mean blood loss was 70.61 ± 60.96 ml. All patients were discharged from the hospital 3 to 8 days after surgery. During surgery, the adrenal gland was slightly lacerated in three cases and the peritoneum showed perforation in six cases. Only one case recurred during the study follow-up.

**Conclusions:**

Based on retroperitoneal laparoscopy radical nephrectomy, this effective adrenal gland-sparing surgery showed direct exposure of tissue and little interference of the upper pole of the kidney. Elevation of the adrenal gland could help with the complete dissection of the adrenal gland from the kidney. The separation of the kidney was rapid, simple and accurate. The probability of adrenal gland damage was reduced. This strategy is recommended for widespread use in T1-2 renal neoplasms.

## Background

Renal cell carcinoma (RCC), accounting for 3% of adult solid tumors, is mostly detected in patients 50 to 70 years old [1]. Fortunately, due to the development of imaging modalities such as ultrasound, computed tomography (CT) and magnetic resonance imaging (MRI), most of these kidney masses can be detected at an early stage and can be cured by surgery [2,3]. Currently, retroperitoneal laparoscopic radical nephrectomy is suggested as the best therapy for early stage RCC [4].

In recent years, laparoscopic radical nephrectomy (LRN) has been widely used, and the number of reports of complications involved in surgery is rising [5,6]. Briefly, compared with open radical nephrectomy, LRN is associated with a smaller incision; less blood loss, narcotic requirement and complications; shorter hospital stay; and earlier resumption of routine activities [7]. The technical progress of laparoscopic surgery for RCC has been remarkable. Laparoscopic partial nephrectomy (LPN) is also widely used, having advantages that include decreased bleeding and speedy recovery [8]. This therapeutic schedule is suitable for patients with only one functional kidney [9]. However, a previous study showed that LRN was superior to LPN because of the lower incidence of complications and higher survivability [10]. In this study, LRN was suitable and chosen for all patients. The retroperitoneal laparoscopic approach to the kidney offered minimally invasive access without violation of the peritoneal cavity [11]. Over the last two decades, all renal surgeries have been shown to be feasible when incorporating this technique. However, this method can include complicated procedures, such as a donor nephrectomy and radical nephroureterectomy, and has developed a number of modifications to make surgery easier and cost effective [12,13]. For the detection of small tumors with less lymph node and adrenal gland involvement [3], there was a tendency to perfect a retroperitoneal laparoscopic approach, which has been proposed as suitable for kidney tumors.

Although some researchers have suggested that adrenalectomy should be performed as an integral part of the surgery [14], modern cases have not shown a benefit to routinely removing the adrenal gland with radical nephrectomy, and the incidence rate of adrenal metastasis is low [15]. In humans, the right adrenal gland is triangular shaped, while the left adrenal gland is semilunar shaped. The adrenal glands affect kidney function through the secretion of aldosterone. Metastasis of RCC to the contralateral adrenal gland is very rare and adrenal metastasis is rarely diagnosed during life [16]. The ipsilateral adrenalectomy during radical or partial nephrectomy does not improve survival; moreover, Brian *et al*. found that there was no significant difference in survival rate between patients with and without adrenalectomy [17]. Adrenal gland-sparing surgery is usually accompanied by adrenal gland tears, which may be due to the separation of the adrenal gland from the kidney. No study has been published on adrenal gland-sparing during retroperitoneal laparoscopic radical nephrectomy.

In the present study, the anatomic relationship between the kidney and adrenal gland was reviewed, and a modified adrenal gland-sparing surgery was explored based on retroperitoneal laparoscopic radical nephrectomy for patients who did not need to have their adrenal gland removed.

## Methods

Our study was approved by the Ethics Committee of Jinling Hospital, Medical School of Nanjing University, Nanjing, China. Written informed consent was obtained from all patients.

### Clinical information

From June 2010 to October 2012, a total of 68 patients suffering from localized RCC were treated at our hospital, including 45 male and 23 female cases, and their average age was 54.06 years. The mean tumor size was 4.7 cm (range, 1.6 to 8.0 cm). Preoperative CT examination excluded local lymph nodes and distant metastasis. The stage for RCC was recorded based on the 2010 7th edition of the American Joint Committee on Cancer (AJCC) TNM staging system [18]. Of the 68 tumors, 57 tumors were pathologic stage T1 and 11 were stage T2. Examination with intraudio videoenous urography (IVU) and emission computed tomography (ECT) verified normal function of the contralateral kidney. If the tumor was located in the lower-middle part of kidney or was a small renal carcinoma, adrenal gland-sparing was needed. Patients with a large tumor or with lymph node or distal metastases were excluded from our study.

### Operative techniques

#### Establishment of retroperitoneal space

Patients were administered general anesthesia via tracheal intubation and were placed in a lateral position in order to elevate the kidney. A mid-axillary line incision was made at two transverse fingers above the crista iliaca. A 10-mm trocar puncture was directed through the incision, pushing the peritoneum ventrally. A lactoprene balloon was placed, injected with 300 ml air (maintained 5 min), and then removed. The 5-mm and 10-mm trocars were inserted in the subcostal incision on the anterior and posterior axillary lines, respectively. Then, CO_2_ was injected into the retroperitoneum to establish a retroperitoneal space (air pressure: 12 to 15 mm Hg), and the corresponding surgical instruments were placed. For obese patients, extraperitoneal adipose tissues were resected using the ultrasonic scalpel to facilitate operation.

### Dissection of kidney

The dissection was performed along the anterior surface of the psoas muscle and fascia to access the connective tissue platform, which can help to provide adequate spacing. The posterior surface of Gerota’s fascia was opened close to the diaphragm to expose the fatty tissue harboring renal vessels. The renal artery was bluntly separated via aspirator and angle clamps and was clipped with Hem-O-Lock. After the structure of the renal vein and the color of kidney were examined, the parenchyma was dissociated to confirm there was no ectopic artery. Then the renal vein was ligated with Hem-O-Lock and the blood flow was blocked.

### Adrenal gland-sparing surgery

**Figure 1:**
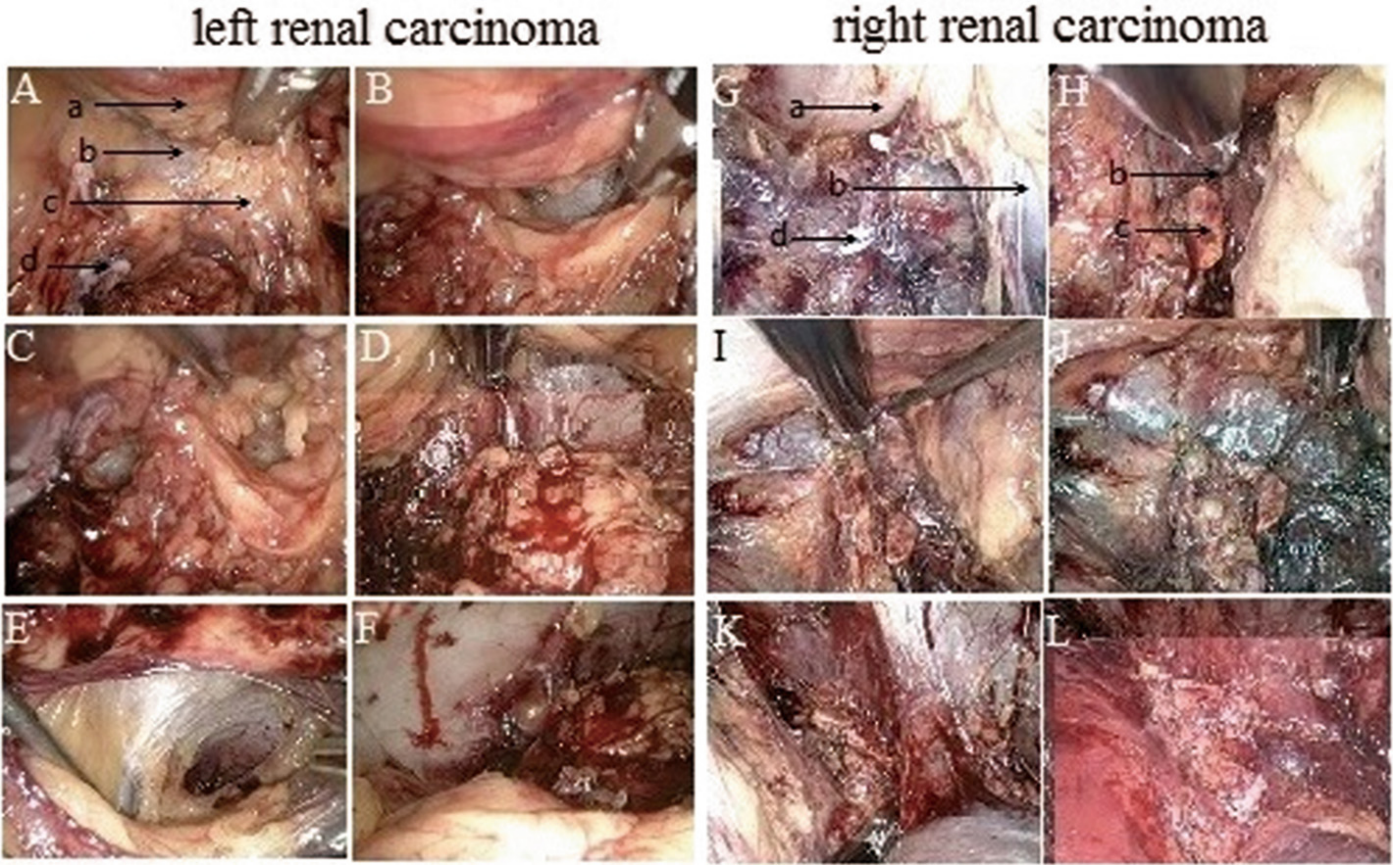
**Radical resection of renal carcinoma located on left side and right side. A-F** represents surgery to the left side. **G-L** represents surgery to the right side. **A** and **G**. Dissection of the interior side of the renal hilum toward the perirenal fascia. **B** and **H**. Dissection of the bottom of adrenal gland toward the perirenal fascia. **C** and **I**. Dissection of the upper pole of the kidney. **D** and **J**. Dissection of the kidney from adrenal gland. **E** and **K**. Complete separation of the kidney from the adrenal gland. **F** and **L**. A relatively intact adrenal gland. a. The upper pole of the kidney. b. The perirenal fascia. c. The adrenal gland. d. The renal pedicle.

**Figure 2:**
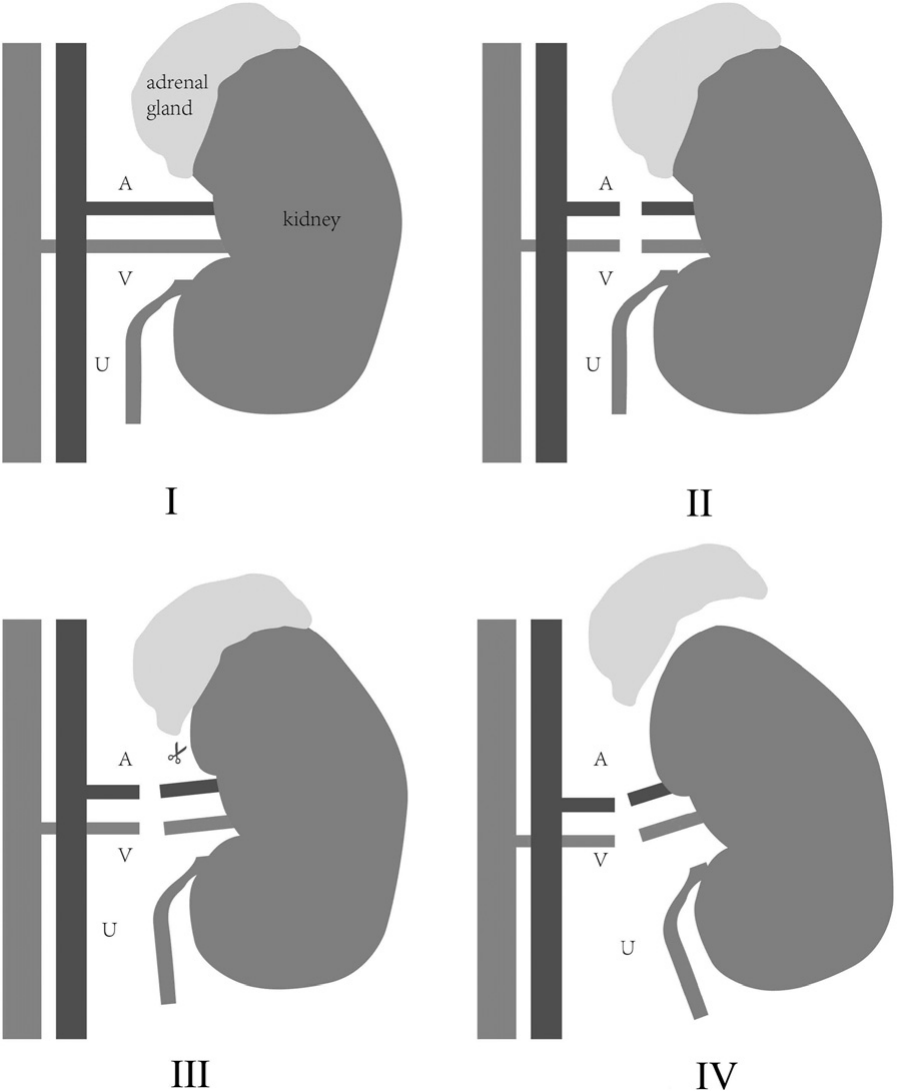
**A model diagram of the adrenal gland-sparing surgery on a right kidney. I**: The anatomic relationship between the kidney and the adrenal gland; **II**: Division of the artery and the vein; **III**: Separation of the kidney from the adrenal gland; **IV**: The intact adrenal gland and the removed kidney. A, artery; V, vein; U, ureter.

The adrenal gland-sparing surgery of a tumor located on the left kidney (Figure 1A-F) was similar to that for a tumor on the right side (Figure 1G-K).After disconnection of the renal pedicle vessels, the interior surface of the perirenal fascia was mobilized from the interior side of the renal hilum and the undersurface of the adrenal gland (Figure 1A,G). After cutting the renal hilum vessels, the adrenal gland was elevated to 1 to 2 cm and was kept with a sufficient tension to provide adequate space. The bottom of the adrenal gland was dissociated along the edge of the renal parenchyma from the posterosuperior pole of the kidney (Figure 1B,H) until the interior pole of the adrenal gland was mobilized (Figure 1C,I). To separate the kidney from the adrenal gland, the dissociation must be close to the perirenal fascia. The inner side of the kidney was further mobilized along the lateral branch of the adrenal gland (Figure 1D,J). The kidney was further mobilized from the perirenal fascia (Figure 1E,K) and completely divided from the parenchyma (Figure 1F,L). The incised kidney and surrounding tissues were placed into the extraction bag. The fascia was closed with interrupted sutures, and the skin incision was closed with buried suture after a drain tube was placed.A model diagram figuratively shows the procedure of the adrenal gland-sparing surgery (Figure 2).

### Postoperative therapy

Antibiotics were administered 1 to 2 days after surgery to prevent infection. The drain tube was removed 1 to 3 d after surgery. For patients with RCC confirmed via postoperative pathology, interferon plus interleukin immunotherapy was administered.

### Follow-up study

Patients were reexamined at 3, 6, and 12 months after surgery. Then, all cases were required to recheck once a year. The examination index included routine blood and urine tests, hepatic and renal function, chest radiograph and abdomen B ultrasonography and CT examination.

## Results

### Clinical characteristics

All patients were successfully operated without conversion to open surgery. The surgical procedure was shown in Figure 1 and Figure 2. The average operative time was 56.65 min (range, 28 to 164 min), and the average estimated bleeding was 70.61 ml (range, 15 to 300 mL). The mean serum creatinine level was 75.47 μmol/l before operation and 94.57 μmol/l after operation. The recovery eating time ranged from 15 to 65 h. Patients were allowed to get out of bed 13 to 60 h after the operation and were discharged to home at 3 to 8 d after operation.

However, in six cases, the peritoneum was perforated and treated with laparoscopic suturing technique during the surgery. Additionally, in three cases, the torn edges of the lateral branch of the adrenal gland were effectively treated with bipolar coagulation hemostasis. No other complication was identified.

### Pathology

Of the 68 tumors, 57 tumors were pathologic stage T1 and 11 were stage T2. Histological analysis of the 68 specimens revealed 61 clear cell tumors, three papillary tumors, one chromophobe tumor, one spindle-cell tumor, one myogenic tumor and one tumor related to Xp11.2 translocation/TFE3 gene fusion (Table 1).

**Table 1:** Clinical information of 68 patients with local renal carcinoma

**Patient demographics (n = 68, mean ± standard deviation)**	
Gender (Male/Female)	45/23
Tumor location (right/left)	35/33
Age (years)	54.06 ± 13.34
Tumor Size (cm)	4.7 ± 1.35
Operation time (min)	56.65 ± 26.60
Blood loss (ml)	70.61 ± 60.96
Time for dieting (h)	27.78 ± 9.26
Time of getting out of bed (h)	31.57 ± 8.79
Hemoglobin (g/l)	
Preoperative hemoglobin	130.38 ± 12.96
Postoperative hemoglobin	118.88 ± 12.86
Serum creatinine (μmol/l)	
Preoperative serum creatinine	75.47 ± 19.32
Postoperative serum creatinine	94.57 ± 33.45
Stage	
T1N0M0	57
T2N0M0	11
Histology	
Clear cell	61
Papillary	3
Chromophobe	1
Spindle cell	1
Myogenic	1
Xp11.2 translocation/TFE3 gene fusion	1

### Follow-up study

**Figure 3:**
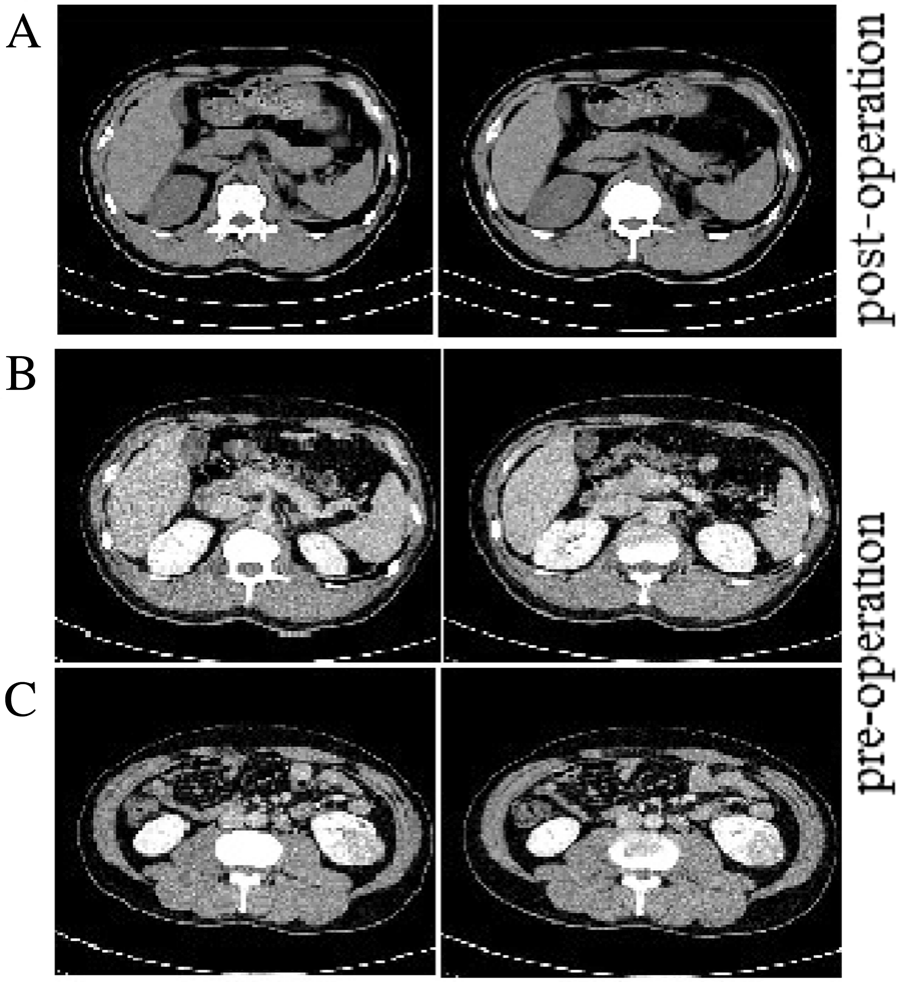
**Computed Tomography (CT) examination of a 43-year-old male with left renal carcinoma. A**. CT imaging at the postoperative check indicated the size, location and integrality of the left adrenal gland. **B**. CT imaging at the preoperative examination showed the relationship between the left kidney and the left adrenal gland. **C**. The preoperative CT imaging displayed the location and size of the tumor.

All patients received injections of interferon plus interleukin for 3 to 6 months after operation. The average follow-up was 18 months (range, 3 to 26 months). Only one case showed lesions on the lateral peritoneum and was further treated via chemotherapy. No local recurrence or distant metastasis was visible via ultrasound and chest X-ray examination, which might be due to the early pathologic stage of all tumors and the short follow-up period. CT examination of one patient with clear cell tumor is shown in Figure 3.

## Discussion

With the growing number of incidentally diagnosed kidney neoplasms, significant advances in the treatment of RCC have been developed during the last 20 years [19]. LRN has been suggested as a safe and effective treatment for renal tumors and has been safely performed for large right-sided T2 tumors [20]. Retroperitoneoscopic LRN surgery must be performed in a narrow operative space and is technically difficult to perform [12]. In recent years, retroperitoneoscopic surgery has been widely used, based on the invention of atraumatic balloon dilation that makes it easy to secure a space [6,21].

Many reports have presented results of comparisons of retroperitoneoscopic surgery with other traditional surgical methods, which have suggested significant technical improvements in retroperitoneoscopic surgery [22,23]. However, the procedure of retroperitoneoscopic surgery, where the main step is the management of the renal pedicle and the adrenal gland, is controversial and needs to improve [24,25]. During the surgery, adrenal gland-sparing is necessary in many cases, such as when tumors are located in the lower-middle part of the kidney, and for small RCC and renal pelvic tumors [26]. Although adrenal gland-sparing is easier than adrenalectomy, lacerations of the adrenal gland usually occurred due to the complex inner structure and even led to wound errhysis or delayed hemorrhage [27]. No reports discussed the adrenal gland-sparing surgery based on LRN and the proper surgery to completely retain the adrenal gland.

In this study, we reviewed the anatomic relationship between the kidney and adrenal gland and discussed a modified adrenal gland-sparing strategy based on retroperitoneal laparoscopic radical nephrectomy. Briefly, the vessels of the renal pedicle were separated; the interior surface of the perirenal fascia was mobilized from the interior side of the renal hilum and the undersurface of the adrenal gland; the adrenal gland was elevated and was kept at a sufficient tension to provide adequate space; the bottom of the adrenal gland was dissociated along the edge of the renal parenchyma; and the kidney was separated from the adrenal gland.

Compared to the traditional process, our adrenal gland-sparing surgery was directly performed on a horizontal plane. The dissection of the adrenal gland from the perirenal fat could reduce the influence of the upper pole of the kidney and the incidence of lacerations or partial resection of the adrenal gland. The upper pole of the kidney could be lifted after separation of the renal pedicle vessels, which could change the tension between the adrenal gland and the kidney and facilitate the dissection of the kidney from the adrenal gland. Other characteristics of our procedure included a small incision of dissection, clear and fixed anatomy, less influence of fat tissues and less variation.

Although the main approach for a left kidney tumor was similar to the right, there were several anatomic differences [28]. For example, the right adrenal central vein converged with the inferior vena cava, while the left adrenal central vein converged into the left renal vein. For the left kidney, dissection must be performed along with the renal pedicle to avoid damage to the left adrenal gland. Furthermore, Dieter *et al*. found that it is beneficial to improve survival by using adjuvant treatment with inhibitors of VEGF-R and mTOR after nephrectomy [29]. Similarly, adjuvant treatment in patients with interferon and interleukin seems to be beneficial and can be considered for administration during the postoperative recovery process.

## Conclusions

In conclusion, adrenal gland-sparing surgery was performed on 68 patients with T1-2 RCC. During the surgery, the peritoneum was perforated in six cases and impaired in three cases. No other complication was identified. Follow-up study showed only one reoccurrence, which was lon the lateral peritoneum. No distant metastasis was visible. This procedure offered smaller incision, shorter operative time and greater outcomes than traditional retroperitoneoscopic surgery. While this study demonstrated that adrenal gland-sparing surgery was a feasible option for small renal masses, its limitations should be stressed. Careful patient and tumor selection criteria should be applied. To further document these results, studies with longer follow-up and other TNM stages are needed.

## Abbreviations

CT: computed tomography; ECT: emission computed tomography; IVU: intraudio videoenous urography; LPN: laparoscopic partial nephrectomy; LRN: laparoscopic radical nephrectomy; MRI: magnetic resonance imaging; RCC: renal cell carcinoma.

## Competing interests

The authors declare that they have no conflicts of interest.

## Authors’ contributions

ZX, WZ and ZZ participated in the design of this study, and they both performed the statistical analysis. JG and JD carried out the study, together with ZW and HT, collected important background information, and drafted the manuscript. XX, CT and XY conceived of this study, and participated in the design and helped to draft the manuscript. All authors read and approved the final manuscript.
